# The Effectiveness of Physical and Chemical Defense Responses of Wild Emmer Wheat Against Aphids Depends on Leaf Position and Genotype

**DOI:** 10.3389/fpls.2021.667820

**Published:** 2021-06-28

**Authors:** Anuradha Singh, Brian Dilkes, Hanan Sela, Vered Tzin

**Affiliations:** ^1^Jacob Blaustein Center for Scientific Cooperation, Jacob Blaustein Institutes for Desert Research, Ben-Gurion University of the Negev, Midreshet Ben-Gurion, Israel; ^2^Department of Biochemistry, Purdue University, West Lafayette, IN, United States; ^3^The Institute for Cereal Crops Improvement, Tel Aviv University, Tel Aviv, Israel; ^4^Institute of Evolution, University of Haifa, Haifa, Israel; ^5^French Associates Institute for Agriculture and Biotechnology of Drylands, Jacob Blaustein Institutes for Desert Research, Ben-Gurion University of the Negev, Midreshet Ben-Gurion, Israel

**Keywords:** benzoxazinoids, defense, phloem sap, *Rhopalosiphum padi*, trichome, wild emmer wheat (*T. turgidum* ssp. *dicoccoides*), electrical penetration graph recording

## Abstract

The bird cherry-oat aphid (*Rhopalosiphum padi*) is one of the most destructive insect pests in wheat production. To reduce aphid damage, wheat plants have evolved various chemical and physical defense mechanisms. Although these mechanisms have been frequently reported, much less is known about their effectiveness. The tetraploid wild emmer wheat (WEW; *Triticum turgidum* ssp. *dicoccoides*), one of the progenitors of domesticated wheat, possesses untapped resources from its numerous desirable traits, including insect resistance. The goal of this research was to determine the effectiveness of trichomes (physical defense) and benzoxazinoids (BXDs; chemical defense) in aphid resistance by exploiting the natural diversity of WEW. We integrated a large dataset composed of trichome density and BXD abundance across wheat genotypes, different leaf positions, conditions (constitutive and aphid-induced), and tissues (whole leaf and phloem sap). First, we evaluated aphid reproduction on 203 wheat accessions and found large variation in this trait. Then, we chose eight WEW genotypes and one domesticated durum wheat cultivar for detailed quantification of the defense mechanisms across three leaves. We discovered that these defense mechanisms are influenced by both leaf position and genotype, where aphid reproduction was the highest on leaf-1 (the oldest), and trichome density was the lowest. We compared the changes in trichome density and BXD levels upon aphid infestation and found only minor changes relative to untreated plants. This suggests that the defense mechanisms in the whole leaf are primarily anticipatory and unlikely to contribute to aphid-induced defense. Next, we quantified BXD levels in the phloem sap and detected a significant induction of two compounds upon aphid infestation. Moreover, evaluating aphid feeding patterns showed that aphids prefer to feed on the oldest leaf. These findings revealed the dynamic response at the whole leaf and phloem levels that altered aphid feeding and reproduction. Overall, they suggested that trichomes and the BXD 2,4-dihydroxy-7- methoxy-1,4-benzoxazin-3-one (DIMBOA) levels are the main factors determining aphid resistance, while trichomes are more effective than BXDs. Accessions from the WEW germplasm, rich with trichomes and BXDs, can be used as new genetic sources to improve the resistance of elite wheat cultivars.

## Introduction

Plants are continuously confronted by various types of herbivorous insects that cause significant yield loss. Aphids (Hemiptera, Aphididae) are phloem-feeder herbivores that consume water and nutrients from their host, reduce plant growth, and transmit devastating plant viruses (Blackman and Eastop, 2000). Plants have evolved physical and chemical defense strategies to control plant-aphid interactions and ensure plant survival and fitness (Mitchell et al., 2016). Constitutive defense mechanisms are present in plant tissues as anticipatory strategies in preparation for forthcoming adverse conditions (Mertens et al., 2021). Defense mechanisms can also be dynamic and induced upon herbivore attack, depending on plant age, developmental stage, and genetic background (Howe and Jander, 2008; Brunissen et al., 2009; Chandrasekhar et al., 2018; Batyrshina et al., 2020a). Variation in these mechanisms at the spatial level (individual parts within a plant) may be one of the key determinants of pest reproduction and feeding behavior (Awmack and Leather, 2002; Karley et al., 2002; Jakobs and Müller, 2018). The relationship between the defense mechanisms, their anticipatory variation, and their effectiveness in response to aphid attack has not been fully addressed.

Physical structures, such as trichomes, epidermal barriers including the cuticle, waxes, and cell wall, and feeding-induced callose deposition, act as the first barriers between the insect and the plant (War et al., 2012). Glandular trichomes are used for exudate storage and secretion, while non-glandular trichomes, specialized epidermal hair-like structures, may affect aphid movement and reproduction rates (Riddick and Simmons, 2014). The leaf surface of young wheat, barley (*Hordeum vulgare*), and tef (*Eragrostis tef*) plants are covered with non-glandular trichomes (Leybourne et al., 2019; de Correa et al., 2020; Gyan et al., 2020). Trichome-based insect resistance is distributed unevenly across plants, tissues, and organs. It is usually more evident in young tissues than in older ones (Valkama et al., 2004; McCall and Fordyce, 2010). Several studies have shown that herbivore feeding induces the subsequently formed leaves to produce higher trichome densities. The inducibility of trichome density is ecologically significant, affecting insect feeding behaviors and limiting their performance in wheat and barley (Roberts and Foster, 1983; de Correa et al., 2020). One chemical defense strategy used in a variety of plants is the production of toxic indole-derived specialized metabolites called benzoxazinoids (BXDs) (Lattanzio et al., 2000; Kettles et al., 2013). These compounds are highly abundant in monocots, such as wheat, maize, rye, and several wild barley species (Frey et al., 2009), and in some distinct dicot families (Schullehner et al., 2008; Hannemann et al., 2018). The abundant BXDs in wheat are DIMBOA (2,4-dihydroxy-7-methoxy-1,4-benzoxazin-3-one), DIMBOA-Glc, and its methylated form HDMBOA-Glc [2-(2-hydroxy-4,7-dimethoxy-1,4-benzoxazin-3-one)-β-d-glucopyranose] (Shavit et al., 2018). The accumulation of these compounds is developmentally variable, but they are usually present in high concentrations in young leaves (Cambier et al., 2000; Batyrshina et al., 2020b). These defensive metabolites can be synthesized *de novo* in response to insect attack and can also be produced constitutively and stored as an inactive form in the vacuole. BXDs possess antifeedant and antibiosis properties (Niemeyer, 1988; Feng et al., 1992; Escobar et al., 1999). For instance, DIMBOA is required for callose formation, which accumulates in response to aphid probing, thus possessing antifeedant characteristics (Yan et al., 1999; Ahmad et al., 2011; Betsiashvili et al., 2015), while HDMBOA-Glc acts as a deterrent to aphids (Meihls et al., 2013), as well as generalist and specialist chewing insects (Glauser et al., 2011).

The mobilization of resources between plant tissues relies on the plant’s vascular system (Griffiths et al., 2016). This process is mediated by the phloem tissue’s sieve elements and associated companion cells, which allow transportation and long-distance distribution of resources. Phloem-feeding insects rely on the plant’s phloem sap composition and water to support their life and reproduction. The phloem cell’s architecture provides an additional line of defense in terms of mechanical barriers, owing to latex exudation, resin, or callose deposition around the sieve elements (Ahmad et al., 2011). The interaction between the aphid and the host plant involves the coordinated reconfiguration of metabolism, which is regulated by developmental, genetic, and environmental inputs (Kooke and Keurentjes, 2011; Batyrshina et al., 2020a). Many defensive compounds, such as glucosinolates, terpenes, and BXDs, have been detected in phloem sap (Chen et al., 2001), which may be particularly effective against aphids and other phloem-feeding insects. The abundance of BXDs in the phloem sap depends on several factors, including (i) biosynthesis of glucosides in the plastid, endoplasmic reticulum membrane, and cytosol, (ii) translocation of glucosides from compartmentalized cells to the vacuoles of undamaged plant cells and activation by specific glucosidases near the damaged sites, and (iii) loading into the phloem (Wouters et al., 2016; Niculaes et al., 2018). Hence, plants can take advantage of BXD biosynthesis and interconversion to improve their defensive strategy at either the leaf tissue or phloem level. To date, most of the studies detailing the function of BXDs in wheat-aphid relationships have focused on BXD composition in the leaf tissue. A few reports have indicated that compounds have also been detected in wheat phloem sap, indicating that their accumulation in sap may be relevant for aphid feeding and resistance (Givovich et al., 1994; Frébortová et al., 2010).

Wheat is a staple crop that provides 20% of human calories and protein nutrition (Shewry and Hey, 2015). At current population growth rates, the demand for food is predicted to increase by 40% by 2050. To meet this need, crop yield must be increased. One of the main reasons for crop loss is pest damage, contributing to 15% of crop losses worldwide (Deutsch et al., 2018). Cultivated wheat has been continuously bred for high yield, but as a result of the various genetic bottlenecks in domestication and breeding, not all alleles contributing resistance to herbivory have been captured from wild relatives. Wild wheat genotypes are adapted to a broader range of biotic and abiotic conditions than cultivated wheat and may contain greater resistance diversity than cultivated species that were developed in a more uniform, protected environment (Huang et al., 2016). Tetraploid wild emmer wheat (WEW) (*T. dicoccoides;* genome BBAA; WEW hereafter) is the progenitor of both durum and bread wheat and is distributed across the Fertile Crescent. Variation in aphid resistance between Triticum species demonstrated the potential of using ancient tetraploid wild emmer to discover novel defense mechanisms (Nevo et al., 2002; Migui and Lamb, 2003). Geneticists and plant breeders can hybridize wild emmer to both tetraploid and hexaploid cultivated wheat and transfer new alleles (Gerechter-Amitai et al., 1984; Peng et al., 1999; Cheng et al., 2010).

In this study, we investigated the effectiveness of wheat physical and chemical defense responses against bird cherry-oat aphid (*Rhopalosiphum padi*), a major insect pest, causing serious economic damage to cereal crops (Rabbinge et al., 1981; Blackman and Eastop, 2000). We hypothesized that aphid resistance is determined by a combination of both BXD levels (chemical defense) and non-glandular trichome density (physical barrier defense). We analyzed the BXDs and trichome densities on the first three leaves (leaf position) for constitutive and aphid-feeding-induced variation. We also investigated variation in phloem sap BXDs and aphid feeding behavior. To sample the diversity in these traits, we first determined aphid reproduction in a diverse panel of 203 accessions of WEW germplasm. Then, we selected a representative subset of eight genotypes for a detailed analysis that spanned the range of aphid reproductive levels in the population, as well as one domesticated durum wheat cultivar. This dataset allowed us to ask several fundamental questions regarding wheat-aphid interactions: (i) Do chemical and physical defenses vary between leaves, and does this affect aphid reproduction? (ii) What is the contribution of aphid-induced versus constitutive resistance mechanisms? (iii) What is the BXD composition of phloem sap, and is this similar to the BXD composition in the whole leaf? (iv) Do these mechanisms contribute equally to the control of aphid reproduction? Addressing these questions has allowed us to identify WEW accessions that can be used as genetic material to improve the aphid resistance of elite wheat cultivars.

## Materials and Methods

### Plant Genetic Material

A panel of 203 accessions of WEW (*Triticum dicoccoides*) that maximized genetic diversity was chosen for screening aphid resistance. This panel was obtained from The Harold and Adele Lieberman Germplasm Bank, The Institute for Cereal Crops Improvement (ICCI), Tel-Aviv University, Israel. Accessions were collected from about 100 locations throughout Israel, and each accession originated from a single plant. The list of accessions and the locations of their collection sites are described in Supplementary Table 1. Additionally, a domesticated tetraploid durum wheat (*Triticum turgidum* ssp. durum) cultivar named Svevo was used (Avni et al., 2014). Svevo was previously characterized for its BXD profile, trichome density, and aphid performance (Chandrasekhar et al., 2018; Shavit et al., 2018; Batyrshina et al., 2020b), and therefore, it was used as a reference genotype in this study.

### Plant Growth and Aphid-Rearing Conditions

Seeds were germinated on Whatman paper soaked in tap water for 48 h before being stored in the dark; then, young seedlings were planted individually in 330-cm^3^ plastic pots filled with moistened soil mix containing >95% organic matter from sphagnum peat moss. Plants of each accession were grown in a randomized block design in the growth room under a controlled photoperiod regime with a 16-h-light/8-h-dark cycle, with an approximate 300 μmol m^–2^ s^–1^ light intensity at a constant 25 ± 2°C temperature. Experiments were conducted on approximately 10-day-old seedlings when 2–3 leaves were merged (Zadoks stage 1.2) (Zadoks et al., 1974). Simultaneously, under similar growth conditions in the insectarium, a colony of the bird cherry-oat aphid (*Rhopalosiphum padi*) was maintained for many generations on 2-week-old barley plants (*Hordeum vulgare* L. cv. Noga). The vitality of the colony was preserved by transferring the colony to fresh non-infested plants every other week.

### Aphid Bioassays

The panel of 203 WEW accessions was screened for aphid reproduction, as was the domesticated tetraploid durum wheat cultivar, Svevo. For aphid bioassay, 10 apterous adult *R. padi* aphids were confined with a fine paintbrush on 10-day-old individual plants, covered with transparent micro-perforated polypropylene bags (15 × 60 cm; Baumann Saatzuchtbedarf, Germany). After 96 h of infestation, aphids on each plant were gently brushed off, and the total number of nymphs and adults were counted from the entire plant (non-choice whole cage bioassay). Due to the large number of WEW accessions, the non-choice whole cage bioassay screening was divided into small batches where the bread wheat “Chinese Spring” genotype was repeatedly used in each batch as a reference. Then, a subset of the WEW was selected, and the total number of nymphs and adult aphids were counted from plant parts (leaves and stem; choice whole cage bioassay). The plant parts (leaf position) were (i) leaf-1: the bottommost and oldest leaf, (ii) leaf-2: the midpoint and middle-aged leaf, and (iii) leaf-3: the topmost and youngest leaf. Aphid counting on an individual leaf was normalized to the average leaf area. The average leaf area was calculated for the three leaves separately for each accession, using the ImageJ software^1^. For evaluating the constitutive (anticipatory) levels, plants were covered with the bags without applying aphids and are referred to as the untreated control. Leaf tissues from untreated and aphid-infested plants were harvested for either trichome counting or rapidly frozen in liquid nitrogen and stored at −80°C for metabolic analysis.

### Non-glandular Trichome Density Analysis

The middle sections of leaf-1, leaf-2, and leaf-3, from untreated and aphid-infested plants, were collected. From each leaf, 1 cm^2^ was excised, cleared with 80% ethanol at 85°C for 15 min, and rinsed with distilled water. The tissue segment was placed on glass microscope slides facing the adaxial side, and the total non-glandular trichomes were counted (Batyrshina et al., 2020b). Images were acquired with a digital camera (Axiocam 305 color) connected to a Zeiss Axioplan 2 Upright Light Microscope (Zeiss, Oberkochen, Germany). For each accession, three images per plant were captured from the middle portion of the leaves, and trichome density in mm^2^ was calculated using ImageJ software (see text footnote 1).

### Benzoxazinoid Extraction and Analysis From Whole Leaves

Approximately 4–6 cm of leaf-1, leaf-2, and leaf-3, from untreated and aphid-infested plants, were cut and immediately frozen in liquid nitrogen to prevent further changes. These samples were referred to as a “whole leaf” fraction. The tissue was ground to a fine powder, weighed, and homogenized with a 1:10 (w:v) benzoxazinoid extraction solution containing 80% methanol, 19.9% DDW, and 0.1% formic acid. For each sample, 10 μg of benzoxazolin-2(3H)-one (BOA; Sigma-Aldrich, United States) from a 1-mg ml^–1^ stock was added. The homogenized samples were vortexed briefly, sonicated for 40 min at 4°C, centrifuged for 5 min at 14,000×*g*, and filtered using a 0.22-μm sterilizing filter membrane (EMD Millipore Corp., Billerica, MA, United States). Approximately 150 μl of the filtered supernatant was transferred to a 200-μl glass insert and placed in a 2-ml HPLC glass vial. Then, 5 μl of the sample was injected, and BXD compounds were separated and detected using a UV-vis detector on a DIONEX UltiMate 3000 high-performance liquid chromatography (HPLC) system equipped with a C18 reverse-phase Hypersil GOLD column (3 μm pore size, 150 × 4.60 mm; Thermo Fisher Scientific, Germany), following the running conditions and data analysis as previously described (Mijares et al., 2013; Shavit et al., 2018). A metabolite with a BXD UV spectrum with a retention time of 8.3 min was identified as either HDMBAO-Glc or HM2BOA-Glc.

### Phloem Sap Collection for Benzoxazinoid Analysis

Wheat plants were grown under controlled growth room conditions for 10 days, then infested with 10 apterous adult *R. padi* aphids for 96 h as described above. Phloem sap from the untreated control and aphid-infested plants was collected from leaf-1 and leaf-2 samples, while leaf-3 (topmost and youngest) was too small and, therefore, not included in this analysis. Phloem sap was collected using an EDTA-facilitated method as described in Tetyuk et al. (2013). In brief, for each sample, eight leaves were excised from the main shoot by cutting at the base of the petiole, and immediately submerged in dishes containing 10 mM of K_2_-EDTA. The cut leaves were gently stacked on top of each other, cut again, and placed in 1 ml of a 10-mM K_2_-EDTA solution for 10–15 min, followed by a thorough wash with deionized water to remove all K_2_-EDTA. The phloem exudates were then collected for 6 h in a new 15-ml tube, containing 3 ml of fresh deionized water, and placed in the light, under similar growth conditions, on a clear box container with wet paper towels at the bottom to allow photosynthesis, maximize humidity, and reduce leaf transpiration. After the intended collection time, the phloem exudates were immediately frozen in liquid nitrogen. Later, 1.5 ml of phloem exudate was dried in a vacuum and resuspended in 100 μl of the BXD extraction solution, and the filtered supernatant was transferred into a 100-μl glass insert and placed in a 2-ml HPLC vial, and BXD compounds were detected as described above.

### Measuring Feeding Patterns Using an Electrical Penetration Graph

The feeding behavior of *R. padi* on selected WEW accessions (TD-805, TD-2056, and Svevo) was monitored via the EPG on a GIGA-8d system (EPG Systems, Wageningen, Netherlands) (Tjallingii and Esch Hogen, 1993). Adult aphids were starved for 1 h prior to wiring. The dorsal surface of the aphids was glued with water-based silver conductive paint to a 2-cm-long gold wire (20 μm in diameter). The experimental setup for EPG recording was performed as previously described (Leybourne et al., 2019). The feeding behavior of aphids on leaf-1 and leaf-2 was monitored, while leaf-3 was excluded due to its small size. The combination of plant accession and specific leaf position was randomized, while data were acquired using the Stylet + d software. New plants and aphids were used in each run, and approximately 18 successful recordings per leaf in each accession were made. Recordings were excluded if aphids spent more than 70% of the recording time in non-probing + derailed stylet + xylem activities, as suggested by Nalam et al. (2020). Waveform recordings were analyzed every 30 s with the EPG analysis software Stylet+ a installed in a computer connected to a Giga direct current amplifier (van Helden and Tjallingii, 2000; Nalam et al., 2018). Different waveform patterns, in which the aphid is engaged in different activities, were identified according to previously described categories (Tjallingii, 1978; Tjallingii and Esch Hogen, 1993). Due to the large number of experimental groups (two leaves from three accessions), aphid behavior was recorded for 3 h to capture only the initial events of significant feeding differences among the accessions (Gyan et al., 2020). Three main phases were analyzed, including (i) epidermis: time until the first probing (t_1Pr), (ii) the total duration of C (s_C), and (iii) the total duration in the phloem E (s_E), which includes phloem salivation (E1) followed by phloem ingestion E2 (s_E1–>E2). Annotated waveforms were converted into time-series data using the Microsoft Excel macros developed by Sarria et al. (2009).

### Statistical Analysis

The statistical analysis in the present study was conducted using Microsoft Excel 2010 and JMP13 software (SAS^2^). The data obtained for aphid reproduction were subjected to a quantile box-plot with continuous fit using the Shapiro–Wilk test for histogram analysis. The effects of leaf position, accession, treatment, and their interaction (leaf position × accession × treatment) were tested using a two-way Analysis of Variance (ANOVA). The differences among the accessions were tested using a one-way ANOVA with Tukey’s honestly significant difference (HSD) test (*p* ≤ 0.05). The statistical differences between the untreated control and the aphid-infested plants for each accession were evaluated using Student’s *t*-test (*p* ≤ 0.05). Multivariate analyses, including Pearson correlations and Principal Component Analysis (PCA) plots, were performed. We measured the effectiveness of overall physical defense (trichomes), chemical defense (sum of all three BXDs), and total defense [each value (trichomes and each BXD)] using median normalized followed by their sum on aphid reproduction. Then a multiple linear regression analysis was performed on the normalized data. GraphPad Prism was used for figure presentation.

## Results

### Variation in Aphid Reproduction in the Wild Emmer Wheat Population

**FIGURE 1:**
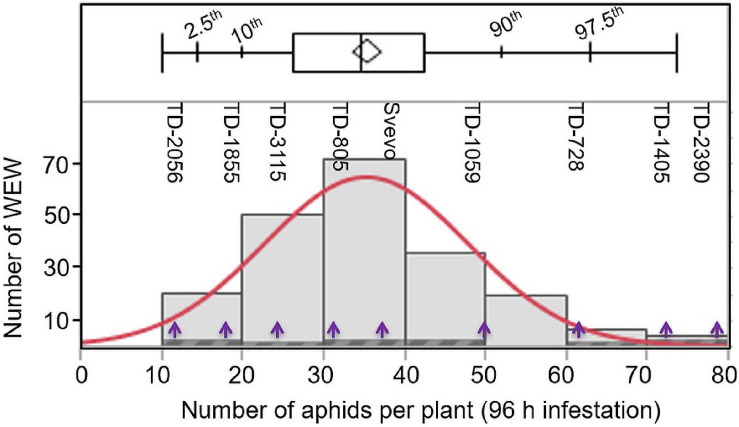
Distribution of 203 wild emmer wheat (WEW) accessions according to their aphid performance. The total aphids per plant (X-axis) were evaluated after 96 h following infestation using a whole cage bioassay (*n* = 8). The distribution ranged from 10 to 80 aphids per plant using the Shapiro–Wilk test shown in red color (*p* < 0.0025). The plot above the histogram displays the quantile box plot with quantile marks (2.5th, 10th, 90th, and 97.5th quantile). The Y-axis represents the number of WEW genotypes in each bin. The purple arrows indicate the nine selected WEW accessions further used in this study.

To determine whether the WEW plants differ in their susceptibility, aphids (*R. padi*) were applied for 96 h on 203 different accessions, and their numbers were recorded. As shown in Figure 1, the number of aphid progeny over the 203 accessions ranged from 10 to 80 aphids per plant, with a median of 34.9 aphids per plant. A few accessions (13.8%; 28 accessions) were found to be highly susceptible, resulting in 50–80 aphids per plant. The number of aphids of the remaining accessions (86.2%; 175 accessions) was ranged from moderately susceptible to resistance with 10–50 aphids per plant. The full list of accessions, including geographic locations and the numbers of aphids on each accession, is presented in Supplementary Table 1. To further understand the resistance mechanism dynamics, a subset of eight WEW accessions was selected from across the range of aphid reproduction including: TD-2056 (10.0; aphids per plant), TD-1855 (13.75; aphids per plant), TD-3115 (24.5; aphids per plant), TD-805 (34.1; aphids per plant), TD-1059 (49.3; aphids per plant), TD-728 (61.5; aphids per plant), TD-1405 (70.4; aphids per plant), and TD-2390 (73.7; aphids per plant). The domesticated durum tetraploid Svevo, with 35.5 aphids per plant, was also included in this subset. These nine genotypes spanned the entire panel distribution, and their positions are marked in Figure 1.

### Leaf Position Affects Aphid Progeny

**FIGURE 2:**
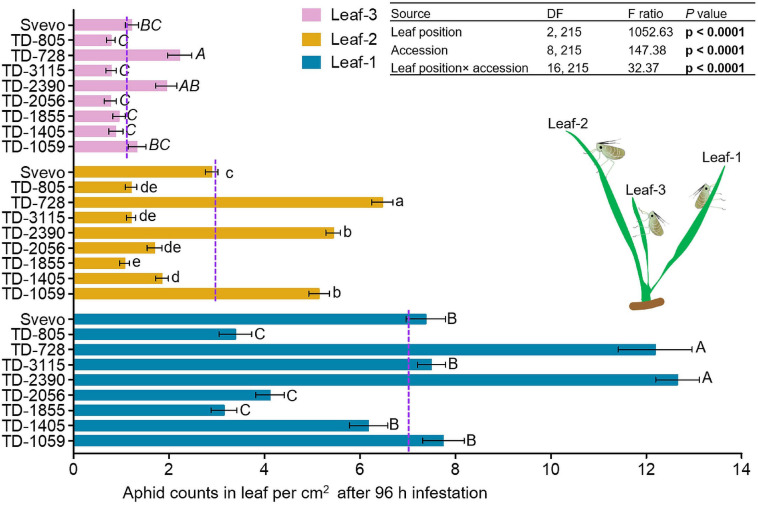
Spatial performance of aphids on selected wheat genotypes. Aphid performance was evaluated in three leaf positions: leaf-1, leaf-2, and leaf-3. The number of total aphids per cm^2^ in each leaf was shown at 96 h following infestation with *Rhopalosiphum padi* using the choice whole cage bioassay (mean ± SE, *n* = 8). The dashed purple lines represent the mean value of aphids per cm^2^ on an individual leaf type among the accessions. The effects of leaf position, accession, and their interaction (leaf position × accession) were tested by two-way analysis of variance (ANOVA) analyses. In bold are the parameters that were significantly affected, *p* < 0.05. Significant differences between accessions are indicated by different letters (one-way ANOVA, Tukey’s honestly significant difference test, *p* < 0.05).

We determined whether leaf position led to differential aphid performances in the selected WEW subset. Therefore, a choice whole cage bioassay was used to evaluate the number of aphids on each leaf. In Figure 2, the numbers of aphids on leaf-1, leaf-2, and leaf-3 are presented. A two-way ANOVA revealed that the aphid number per unit of leaf area was significantly affected by leaf position (*F*_2_,_215_ = 1052.63, *p* < 0.0001). The mean value of aphids per leaf was found to be highest in leaf-1 (7.14), lower on leaf-2 (2.99), and lowest on leaf-3 (1.21). This is consistent with the optimal defense theory, where plant defenses are allocated to younger leaves, which have greater potential to contribute to future fitness (McCall and Fordyce, 2010). There was also a significant effect of wheat accession (*F*_8_,_215_ = 147.38, *p* < 0.0001) and interaction between leaf position and accession (*F*_16_,_215_ = 32.37, *p* < 0.0001) on aphid performance. Overall, this suggests that the leaf position and genetic background affect aphid reproduction in WEW seedlings. This might be the outcome of the differential magnitudes of defense strategies.

### Trichome Density Varies Across Leaf Position

**FIGURE 3:**
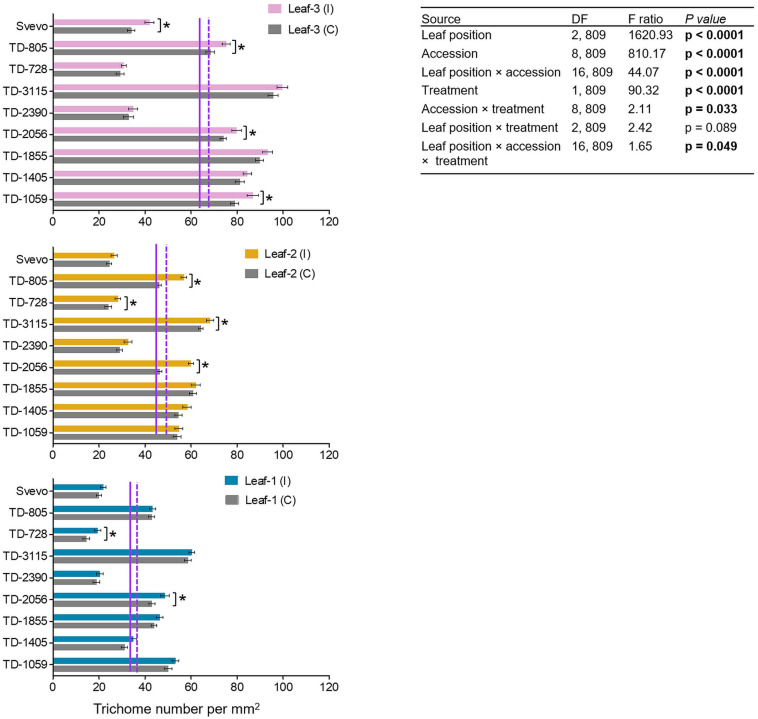
Trichome densities of nine selected wheat genotypes. Trichome density per mm^2^ was evaluated in leaf-1, leaf-2, and leaf-3 in both the untreated control (C) and 96 h following infestation (I) with *R. padi* using the choice whole cage bioassay. Values are expressed as mean ± SE (*n* = 5 replicate with three images per replications). The solid and dashed purple lines represent the mean value of each leaf type under the untreated control and infested conditions among the accessions, respectively. The effects of leaf position, accession, treatment, and their interaction (leaf position × accession × treatment) were tested by two-way ANOVA analyses. The asterisk represents the significant difference between treatments in a particular accession analyzed by Student’s *t*-tests (*p* < 0.05). In bold are the parameters that were significantly affected, *p* < 0.05.

The differential aphid performance on the youngest leaves (leaf-3), as compared to more mature leaves (leaf-1), prompted the quantification of non-glandular trichomes as a physical defense. As presented in Figure 3, the constitutive number of trichomes was the highest in leaf-3 (64.85), lower in leaf-2 (44.79), and lowest in leaf-1 (35.84). Aphid infestation enhanced the trichome density with the strongest effect in leaf-3 (69.63), followed by leaf-2 (49.70) and leaf-1 (38.64). Then, a two-way-ANOVA was used to elucidate the contribution of each parameter: genotype (accession), leaf position (leaf-1-3), and treatment (untreated control and aphid-infested). In the control plants, the trichome density was highly variable, with major significant differences across leaf position (*F*_2_,_809_ = 1620.93, *p* < 0.0001), the nine accessions (*F*_8_,_809_ = 810.17, *p* < 0.0001), and their interaction (*F*_16_,_809_ = 44.07, *p* < 0.0001). The aphid-infested results showed that the trichomes were significantly increased by 6.04–29.13% (*F*_1_,_809_ = 90.32, *p* < 0.0001) upon aphid feeding. The induction was also significant between accessions × aphid infestation treatment (*F*_8_,_809_ = 2.11, *p* = 0.033) and the interaction between accession × leaf position × aphid infestation treatment (*F*_16_,_809_ = 1.65, *p* = 0.049). The leaf position did not interact with treatment (*F*_2_,_809_ = 2.42, *p* = 0.089). Using pair-wise Student’s *t*-tests between the infested and untreated leaves indicated that in four WEW accessions, trichomes were significantly more abundant in leaf-3, while only two accessions were significantly altered in leaf-1. This emphasizes the adaptive plasticity of young leaves to aphid herbivory and the variation in aphid-induced responses.

### Chemical Defensive Compounds: Constitutive and Inducible Benzoxazinoid Levels in the Whole Leaf

**FIGURE 4:**
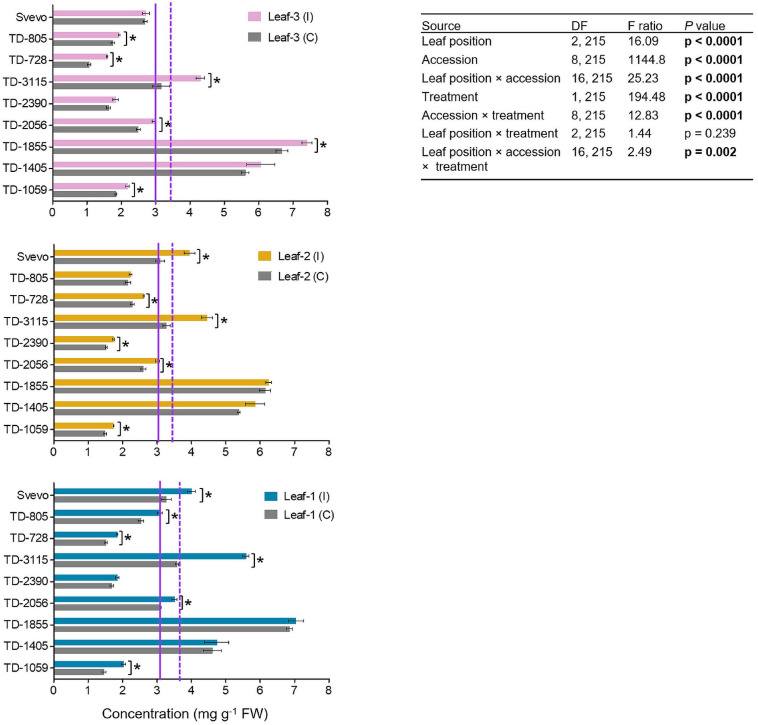
The levels of DIMBOA of nine selected wheat genotypes. The content of the BXD, DIMBOA, (mg per g FW) was evaluated in leaf-1, leaf-2, and leaf-3 in untreated controls (C) and 96 h following infestation (I) with *R. padi* using the choice whole cage bioassay. Values are expressed as mean ± SE (*n* = 4). The solid and dashed purple lines represent the mean value of each leaf type under control and infested conditions among the accessions, respectively. The effects of leaf position, accession, treatment, and their interaction (leaf position × accession × treatment) were tested by two-way ANOVA analyses. The asterisk represents the significant difference between treatments in a particular accession analyzed by Student’s *t*-tests (*p* < 0.05). In bold are the parameters that were significantly affected, *p* < 0.05.

In wheat, BXDs play an important role in chemical defense against a variety of biotic stresses, including insect herbivores (Zhou et al., 2018). Therefore, the abundance of these defensive metabolites was determined in the three leaves. In total, three BXDs, DIMBOA, DIM2BOA-Glc, and HDMBOA-Glc/HM2BOA-Glc, were detected across all nine accessions while DIMBOA was the most abundant BXD in WEW leaf tissues (Figure 4). The WEW accessions, both in the control and aphid-infested plants, had overall average DIMBOA mg g^–1^ fresh weight values that slightly differed across leaf-1 (3.86), leaf-2 (3.71), and leaf-3 (3.51). A two-way-ANOVA was conducted in order to reveal the contribution of each of the three parameters: genetics, leaf position, and treatment. The constitutive DIMBOA levels significantly differed among accessions (*F*_8_,_215_ = 1144.80, *p* < 0.0001), leaf position (*F*_2_,_215_ = 16.09, *p* < 0.0001), and their interaction (*F*_16_,_215_ = 25.23, *p* < 0.0001). Further, the DIMBOA level, in response to aphid treatment, significantly increased from 1.16 to 43.8% (*F*_1_,_215_ = 194.48, *p* < 0.0001). Significant interactions for genotype × aphid treatment (*F*_8_,_215_ = 12.83, *p* < 0.0001) and accession × leaf position (*F*_16_,_215_ = 2.49, *p* = 0.002) were observed. There was no significant interaction between leaf position and treatment (*p* = 0.239). The levels of DIM2BOA-Glc and HDMBOA-Glc/HM2BOA-Glc followed similar spatial patterns as found in DIMBOA. The mean levels of these two compounds were highest in leaf-1, lower in leaf-2, and lowest in leaf-3, across all accessions and both treatments (Supplementary Figures 1, 2). In contrast to physical defenses, which were more pronounced in the youngest leaf, the abundance of chemical defensive compounds was greater in mature leaves.

### Benzoxazinoid Abundance in Wheat Phloem Sap

**FIGURE 5:**
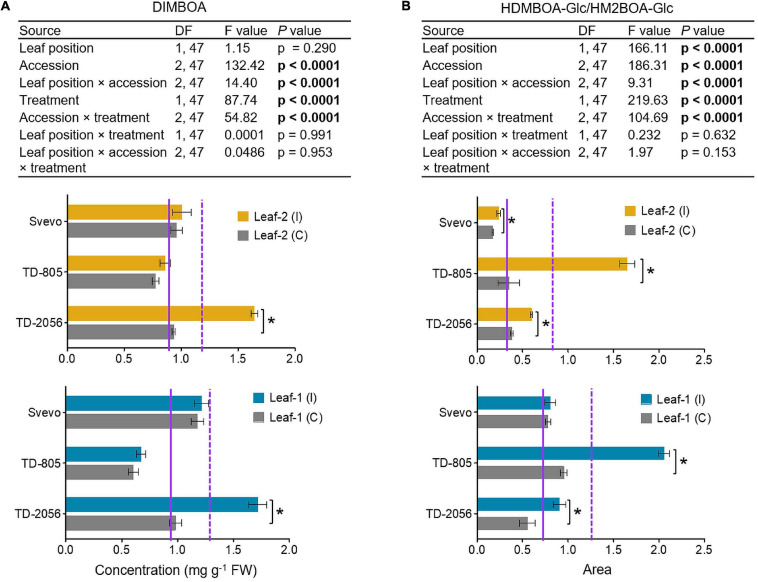
Quantification of chemical defensive metabolites in the phloem sap. **(A)** DIMBOA concentration (mg per g FW) and **(B)** HDMBOA-Glc/HM2BOA-Glc content (peak area) were evaluated in the phloem sap obtained from leaf-1 and leaf-2 in both untreated controls (C) and 96 h following infestation (I) with *R. padi* using the choice whole cage bioassay. Values are expressed as mean ± SE (*n* = 4). The solid and dashed purple lines represent the mean value for each individual leaf type under control and infested conditions among the accessions, respectively. The effects of leaf position, accession, treatment, and their interaction (leaf position × accession × treatment) were tested by two-way ANOVA analyses. The asterisk represents the significant difference between treatments in a particular accession analyzed by Student’s *t*-tests (*p* < 0.05). In bold are the parameters that were significantly affected, *p* < 0.05.

To determine the different BXD abundance levels in the phloem, we measured these metabolites in phloem sap collected from untreated and aphid-infested plants of two selected WEW accessions, TD-805 and TD-2056, and the durum wheat, Svevo. Phloem sap was collected from leaves 1 and 2, but sufficient samples for metabolomic analysis could not be obtained from leaf-3 due to its small size. Only two BXD compounds were detected in the sap, DIMBOA and HDMBOA-Glc/HM2BOA-Glc, and their levels were low relative to the whole leaf (Figures 4, 5). The average of the constitutive DIMBOA level in leaf-1 was 0.92 mg g^–1^ fresh weight, while it was slightly lower in leaf-2, 0.88 mg g^–1^ fresh weight (*p* = 0.290; leaf position). It was strongly influenced by genetic background (*F*_2_,_47_ = 132.42, *p* < 0.0001), where the TD-805 accession accumulated the least amount of DIMBOA. A significant interaction between leaf position × accession was also observed for the DIMBOA level (*F*_2_,_47_ = 14.40, *p* < 0.0001). In response to aphid treatment, the DIMBOA levels increased (*F*_1_,_47_ = 87.74, *p* < 0.0001), and treatment interacted with the accession (*F*_2_,_47_ = 54.82, *p* < 0.0001), with TD-2056 exhibiting the highest increase. No significant effects were observed in leaf position interacting with treatment and/or accessions.

The HDMBOA-Glc/HM2BOA-Glc levels were higher in leaf-1 than in leaf-2, under both the control and aphid-infested treatments (Figure 5B). The basal level of HDMBOA-Glc/HM2BOA-Glc was significantly affected by accession (*F*_2_,_47_ = 186.31, *p* < 0.0001), leaf position (*F*_1_,_47_ = 166.11, *p* < 0.0001), and interaction between these factors (*F*_2_,_47_ = 9.31, *p* < 0.0001). The levels of this compound significantly increased upon aphid treatment (*F*_1_,_47_ = 219.63, *p* < 0.0001), where all the accessions were significantly affected (*F*_2_,_47_ = 104.69, *p* < 0.0001), but not with leaf position (*p* = 0.632) or the interaction between leaf position × accession × treatment (*p* = 0.153). The HDMBOA-Glc/HM2BOA-Glc level increased in TD-805 and TD-2056 upon aphid feeding.

### The Effect of Leaf Position on Aphid Feeding Behavior

**FIGURE 6:**
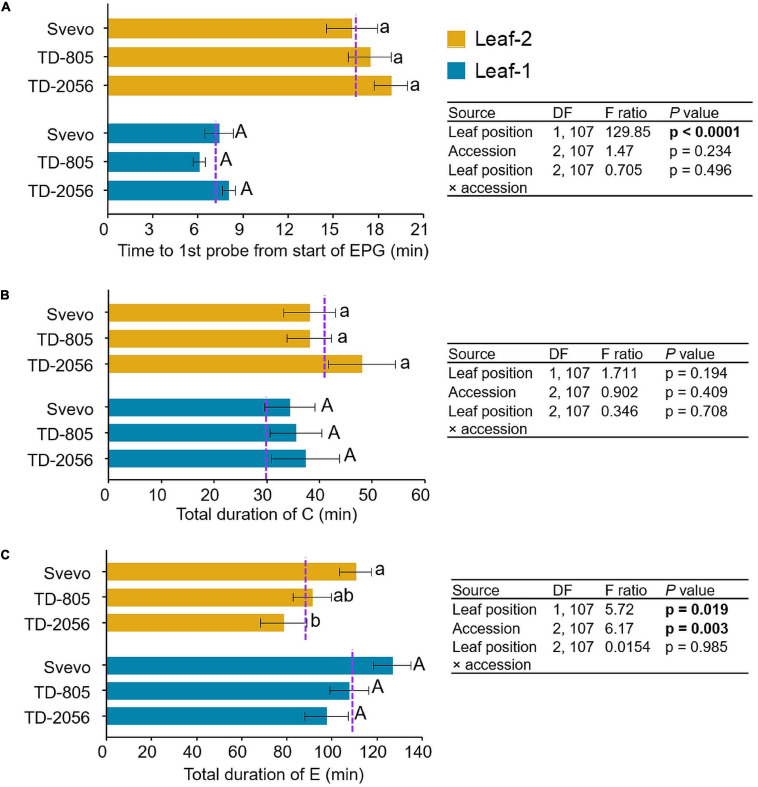
Feeding behavior of *R. padi* aphids. Aphid feeding was measured on leaf-1 and leaf-2 of two selected wild emmer wheat (WEW) accessions and one domesticated durum wheat cultivar. Data of electrical penetration graph (EPG) waveforms were recorded in minutes. **(A)** time until first probe from the start of EPG, **(B)** total duration of C, and **(C)** total duration of E. Values are expressed as mean ± SE (*n* = 18). The dotted purple line represents the overall mean value of the waveform on individual leaf types among the accessions. The effects of leaf position, accession, and their interaction (leaf position × accession) were tested by two-way ANOVA analyses. Significant differences between accessions are indicated by different letters (one-way ANOVA, Tukey’s honestly significant difference test, *p* < 0.05). In bold are the parameters that were significantly affected, *p* < 0.05.

To determine the influence of variation in defense mechanisms on aphid behavior, *R. padi* feeding behavior was examined. We used an electrical penetration graph (EPG) technique to compare the aphid feeding patterns on leaf-1 and leaf-2 among three selected accessions. As shown in Figure 6, the comparisons of the feeding behavior revealed a significant difference in the time until the first probe from the start of EPG between leaf-1 and leaf-2 (*F*_1_,_107_ = 129.85, *p* < 0.0001). Aphids waited longer until their first probing in leaf-2 (17.48 min) than leaf-1 (7.16 min), with no significant difference between accessions (*p* = 0.234) or the interaction between them (*p* = 0.496). In contrast, the time spent in the pathway phase was not significantly altered by leaf position (leaf-1; 35.71 min, and leaf-2; 41.31 min, *p* = 0.193), accession (*p* = 0.409), or the interaction between them (*p* = 0.708). The aphids’ feeding duration in the phloem phase was much longer (101.91 min) than in the other measured phases. Aphids spent significantly more time in the phloem phase on leaf-1 (110.5 min) than on leaf-2 (93.3 min) (*F*_1_,_107_ = 5.72, *p* = 0.019). The feeding time also differed significantly between the accessions (*F*_2_,_107_ = 6.17, *p* = 0.003). The time spent by aphids in the salivation E1 phase did not significantly differ between leaf-1 (3.8 min) and leaf-2 (4.4 min) (*p* = 0.723) and accession (*p* = 0.08). The subsequent E2 phase was longer (>10 min), and aphids spent significantly more time in E2 on leaf-1 (106.7 min) than on leaf-2 (88.9 min) (*F*_1_,_107_ = 5.629, *p* = 0.019) with a significant difference between the accessions (*F*_2_,_107_ = 4.008, *p* = 0.021), as presented in Supplementary Figure 3. Overall, the EPG analysis of *R. padi* aphids showed a clear and significant difference in feeding performance between leaf positions through the differences in total time until first probing and the total duration of E (specifically E2), whereas time spent during the C phase and the E1 phase differed only between accessions. Furthermore, only the total duration of E exhibited a significant interaction between accession and leaf position and not any other EPG event waveform.

## Discussion

### *Rhopalosiphum padi* Aphids Prefer to Feed on Old, Less Protected Wheat Leaves

Our results indicated that both BXD levels and trichome density depend on the leaf position. While the trichome density was highest in the younger leaf (leaf-3) and the lowest in the oldest leaf (leaf-1), the BXD levels showed the opposite trend (Figures 3–4, respectively). Each strategy operates under spatial constraints, in which trichomes rely on young tissues’ more plastic cell division and morphogenesis, and BXD accumulation is dependent on biochemical activities. The BXDs belong to a diverse class of specialized metabolites that play a crucial role in plant resistance to insects (Klun et al., 1967; Argandora et al., 1981; Cambier et al., 2001; Dafoe et al., 2011; Glauser et al., 2011), in arresting fungal infection (Oikawa et al., 2004), and in allelopathy affecting weed growth (Niemeyer, 2009), and they also function in shaping the root microbiome (Neal et al., 2012; Kudjordjie et al., 2019), and iron chelators in maize (Hu et al., 2018). Trichomes serve as a barrier against various external factors, including herbivores and pathogens, UV-B radiation, extreme temperatures, and excessive water loss. They also act as a mechanosensory switch, transducing mechanical stimuli into physiological signals (Werker, 2000; Liu et al., 2016; Fambrini and Pugliesi, 2019). The abundance of chemical and physical defenses depends on several factors, including variety, tissue, and age, where they are mostly high in young seedlings and tend to decline during development toward the juvenile stage (Ebisui et al., 1998; Cambier et al., 2000; Nomura et al., 2005, 2008). In maize, for example, the levels of DIMBOA-Glc and DIM2BOA-Glc were the highest 10 days after seed germination (Cambier et al., 2000). Trichome density is also age-dependent, reported as being high in young leaves and decreasing with leaf expansion (Pérez-Estrada et al., 2000). In the late stages of leaf development, when the formation of the epidermis is completed, trichomes’ functional roles become less important, and leaves often senesce and shed (Valkama et al., 2004). Notably, in our recent reports, we compared the aphid resistance of wheat genotypes in a lab-controlled growth room, counting aphid reproduction after 96 h on wheat seedlings (Batyrshina et al., 2020b), with an evaluation of the natural aphid population on 3-month-old plants grown in the field (Batyrshina et al., 2020a). We found the opposite trends between the two growth conditions, where Svevo was more resistant in the lab conditions and more susceptible in the field versus the WEW accession, Zavitan. Therefore, we suggest expanding this experiment and testing selected WEW accessions in the field across various plant developmental stages, and in the lab with diverse aphid infestation durations.

### The Constitutive Levels of the Trichomes, DIMBOA, and DIM2BOA-Glc Are the Main Factors That Determine Aphid Performance

**FIGURE 7:**
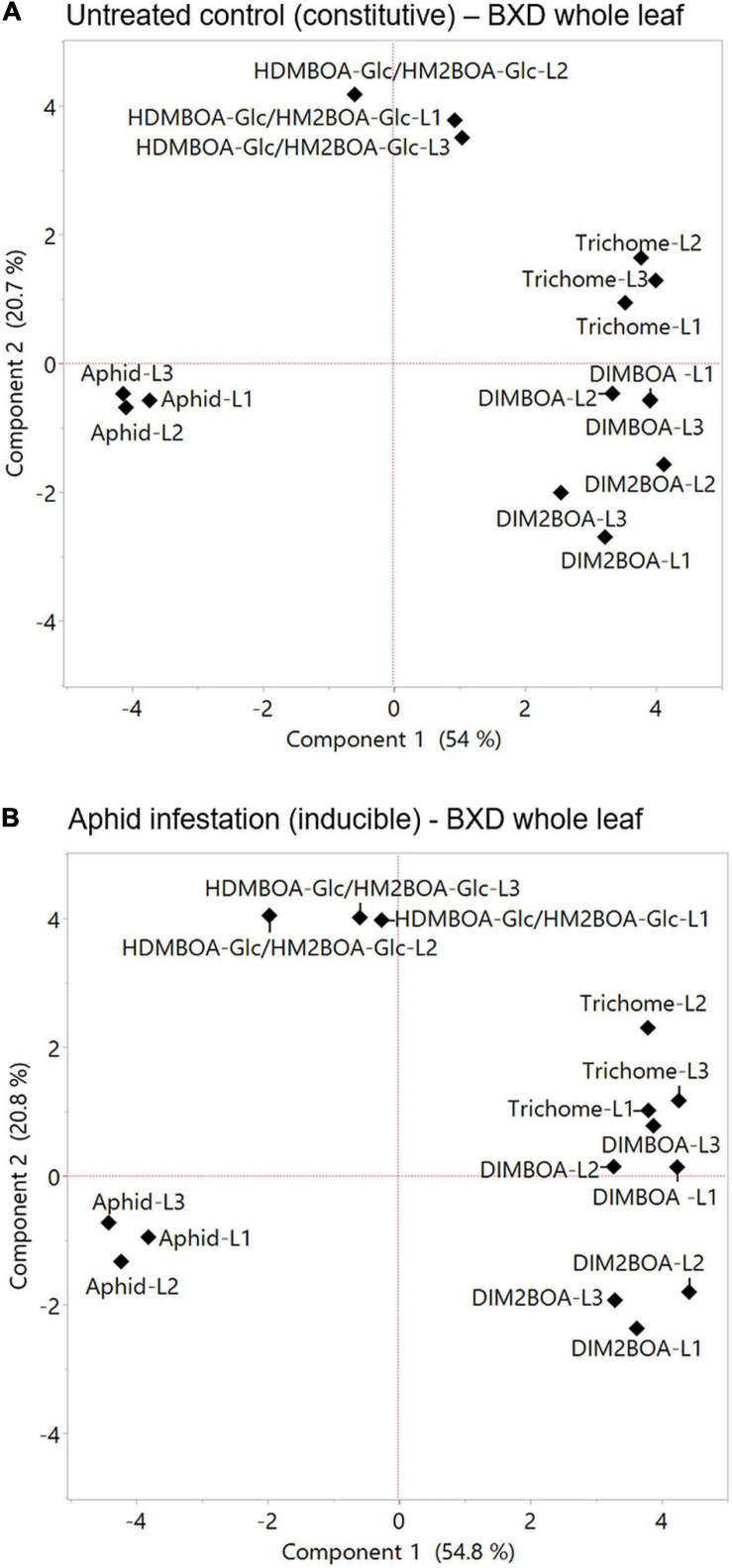
Principal component analysis plots of plants’ physical and chemical defense parameters measured at the whole leaf level. The relation between trichome density, BXDs of the three leaves (leaf-1 [L1], leaf-2 [L2], and leaf-3 [L3]) under untreated control **(A)**, and aphid infestation **(B)** on aphid reproduction. The data for the aphid bioassay, trichomes, and BXDs were normalized to log2 transformation for the projection of PCA. The accessions that were tested in this analysis are TD-728, TD-805, TD-1059, TD-1405, TD-1855, TD-2056, TD-2390, TD-3115, and Svevo, the domesticated durum wheat.

Plants respond to herbivory through multiple morphological, biochemical, and molecular mechanisms. These mechanisms are wide-ranging between plant species and are either constitutively present or induced in response to damage (War et al., 2012). Defense strategies can be affected by different factors, such as developmental stages, tissue, leaf position, genetics, and the perception of environmental cues, which, taken together, govern the potential for aphid resistance (Cambier et al., 2000). To assess the relationships between various mechanisms and aphid progeny on constitutive and inducible levels, PCAs were conducted (Figures 7A,B, respectively). The results indicated that DIMBOA, DIM2BOA-Glc, and trichomes were grouped opposite to the aphid performance, while HDMBAO-Glc/HM2BOA-Glc levels measured in the three leaves were clustered separately. The negative relationship between aphids and DIMBOA might be due to its dual role in defense as both: (i) a deterrent molecule and (ii) a cell-wall-mediated defense strategy affecting callose deposition (Betsiashvili et al., 2015). Both leaf trichomes and BXDs that protect plants from insect herbivory are constitutively produced and can also be induced in response to biotic stresses (Traw and Bergelson, 2003). The constitutive levels of trichome density are negatively related to aphid progeny (Batyrshina et al., 2020b), as well as the increase in the trichome density on new leaves (Traw and Bergelson, 2003). It was previously reported that aphid progeny was negatively correlated with the anticipatory levels of DIMBOA-Glc and positively correlated with HDMBOA-Glc (Meihls et al., 2013). HDMBOA-Glc/HM2BOA-Glc and DIMBOA-Glc showed only a minor induction, depending on different wheat or aphid species (Shavit et al., 2018). To determine the contribution of the inducible defense mechanism to aphid resistance, we compared the parameters of the two PCAs, shown in Figure 7A (constitutive levels) and Figure 7B (inducible levels). The correlation coefficient of comparing between the eigenvectors of PC1 and PC2 revealed a very high similarity between the values (*r* = 0.982 for Component 1 and *r* = 0.972 for Component 2), as presented in Supplementary Figure 4. The results emphasize that only minor changes occur in the defense mechanisms after aphid feeding for 96 h. Thus, we suggest continuing the search for aphid resistance mechanisms by focusing on the anticipatory levels, which can conserve the amount of work and resources invested in this intensive screening.

### Trichome Density Is a More Effective Defense Than BXDs

Although many reports have studied the factors that influence aphid resistance, the relationship between the factors and their effectiveness is unclear. To determine the effectiveness of plant defenses against aphids, we performed multiple linear regression analyses. As shown in Supplementary Figure 5, the predictors–trichome density (physical defense), total chemical defense (sum of BXDs at leaf level), and total defense–explained 46.9% (adjusted *R*^2^) of aphid resistance. Among them, trichome density was the most powerful, with a higher magnitude of aphid resistance (*p* < 0.0001) than chemical defense (*p* = 0.0067). The integration of physical defense into the chemical defense improved the prediction of aphid resistance (*p* = 0.0047), suggesting that physical defense is more effective than chemical defense.

**FIGURE 8:**
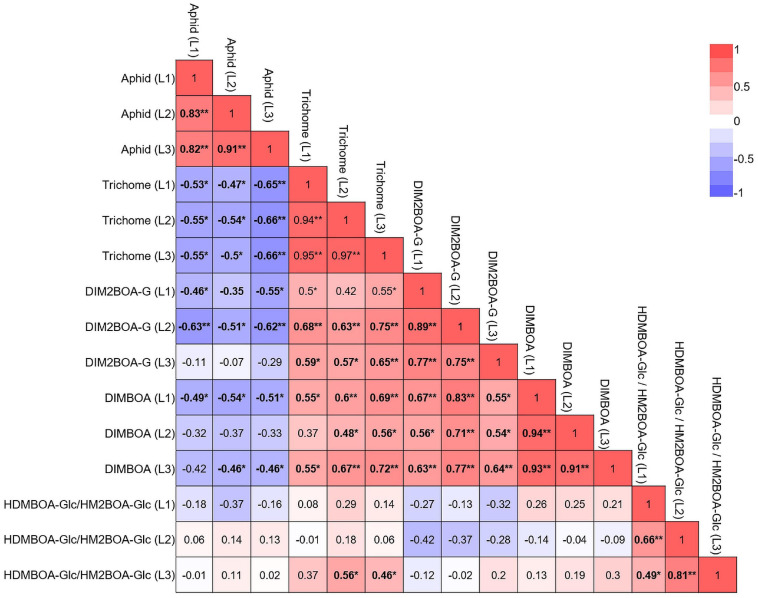
Multivariate correlation between the plants’ physical and chemical defense. The constitutive and inducible data for plant defense were pooled together for correlation analysis. Red indicates a positive correlation, and blue indicates a negative correlation. *p < 0.05; **p < 0.01.

We also correlated all parameters in each leaf, both constitutive and inducible, as presented in a heatmap. These results, shown in Figure 8, indicated that aphid progeny in the three leaves was negatively correlated with trichomes, DIMBOA, and DIM2BOA-Glc. Trichome density was the only parameter that was significantly negatively correlated with aphid progeny in all three leaves. DIM2BOA-Glc was only negatively correlated in leaf-1 and leaf-2, and DIMBOA was negatively correlated in leaf-1 and leaf-3. HDMBOA-Glc/HM2BOA-Glc had no significant correlation with aphid progeny. The non-glandular trichomes on the leaf surface can interrupt the stylet insertion of phloem feeders (Handley et al., 2005; Sato and Kudoh, 2015). The feeding behavior results emphasize that leaf-2’s high number of trichomes might have extended the time for aphid penetration to the leaf mesophyll (Figure 6A). In our previous study, we quantified the BXD levels and trichome density of wheat seedlings from three genotypes, Svevo, Chinese Spring, and a WEW accession named Zavitan (Batyrshina et al., 2020b). These data suggested that in domesticated wheat, the BXD levels provide a better defense mechanism than trichomes against *R. padi* aphids, while Zavitan possessed high trichome density and mild susceptibility. This can be due to differences in sample sizes and genetic diversity, as well as conducting measurements on different leaves. We concluded that in these selected WEW accessions, trichomes are the main factor determining aphid reproduction, while the BXDs may have more complicated regulation and distribution across the genotypes and leaf position. Unlike trichomes, BXDs are synthesized in the leaf and mobilized in the phloem sap. Their effect on aphids depends on their abundance in the phloem and other tissues penetrated by these insects on their way to the phloem. DIMBOA-Glc was also found in the apoplast of maize leaf and increased upon aphid infestation (Ahmad et al., 2011), which may reflect the complexity of the link between BXDs and defense.

### The Benzoxazinoids Have Different Abundance Levels in the Phloem Sap Than in the Whole Leaf

**FIGURE 9:**
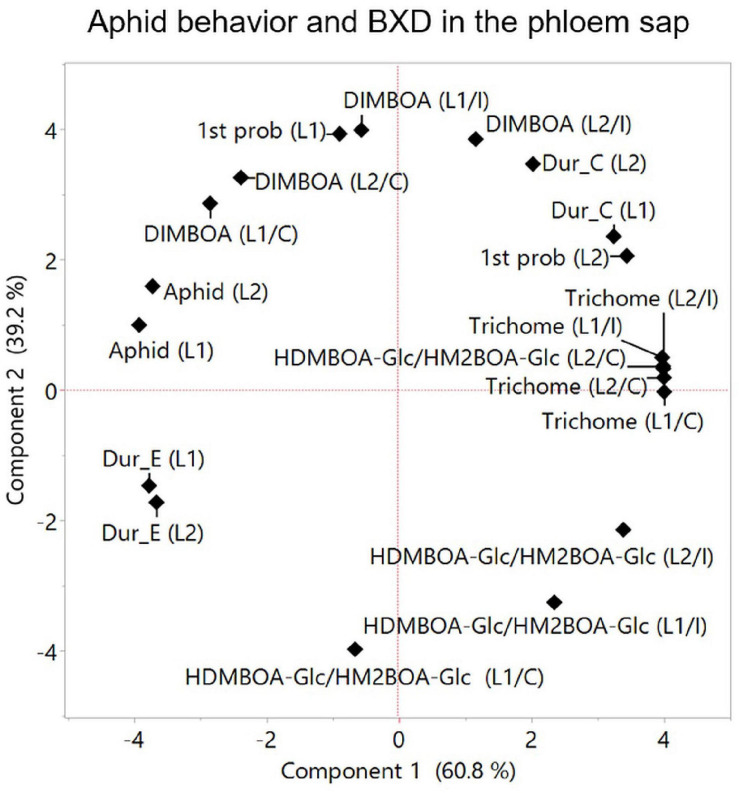
Principal component analysis plot for parameters measured at the phloem level. Aphid feeding behavior was analyzed using the EPG technique only for two leaves (leaf-1 [L1] and leaf-2 [L2], and results were integrated to aphid progeny, trichome density, and BXDs (DIMBOA and HDMBOA-Glc/HM2BOA-Glc) detected in the phloem sap. The data for the trichomes and BXDs are the constitutive levels [C] and aphid-induced [I]. Average values were normalized to log2 transformation for the projection of PCA. The accessions that were tested in this analysis are TD-805, TD-2056, and Svevo.

Aphids solely feed on phloem sap, therefore the BXD composition in the phloem directly affects their fitness. BXD abundance levels in the phloem sap depend on several factors, including (i) biosynthesis in the leaves, (ii) translocation from compartmentalized vacuoles and aglycone activation, and (iii) transportation in the phloem (Wouters et al., 2016; Niculaes et al., 2018). Aphids can sense aglycones BXD on the leaf tissues (Wouters et al., 2016). Prior to feeding, the aphid’s stylet penetrates the plant’s epidermis and passes through the apoplast, where BXD glucosides are present, which exhibit antifeedant activity (Hewer et al., 2011; Schwarzkopf et al., 2013). The aphid stylet’s penetration into the sieve elements leads to the hydrolysis of BXD glucosides and produces a locally high concentration of toxic BXD against herbivores (Wouters et al., 2016). Therefore, the allocation of different BXDs via phloem tissues allows the dynamic protection of plants. In the present study, two BXD metabolites, DIMBOA and HDMBOA-Glc/HM2BOA-Glc, were detected in the phloem sap, while in the whole leaf, DIM2BOA-Glc was also detected. The basal level of DIMBOA in the phloem did not differ greatly among the leaf positions within the accessions. However, a high induction of DIMBOA and HDMBOA-Glc/HM2BOA-Glc levels was found among the different leaf positions from the WEW accessions, suggesting that plants can opt for either biosynthesis or transport activity for BXDs in phloem tissues (Givovich et al., 1994). Recently, two transporter systems of ATP-binding cassette (ABC) transporters and multidrug and toxic compound extrusion transporters (MATE) (Baetz and Martinoia, 2014) were found to play a role in the release of antifungal or antimicrobial root exudates (Nawrath et al., 2002; Stukkens et al., 2005; Bienert et al., 2012). The connection between these transporters and BXD allocation in root cap border cells (Niculaes et al., 2018) or phloem sap is still unclear and requires further investigation. The HDMBOA-Glc/HM2BOA-Glc levels in the phloem sap from leaf-2 displayed a negative effect on aphid performance, while the DIMBOA level displayed a positive effect (Figure 9). This was supported by a previous report that HDMBOA-Glc is more toxic to *R. maidis* aphids than DIMBOA-Glc when administered in an artificial diet (Meihls et al., 2013). HDMBOA-Glc/HM2BOA-Glc in leaf-2 might have had toxic properties, as was explained by the feeding behavior measurements that showed a positive association with the time spent in the path phase (C phase). DIMBOA showed a positive association with aphid progeny (Figure 9), which was in contrast to a previous report where BXDs in the phloem sap of three bread wheat cultivars showed a negative correlation between DIMBOA-Glc and aphid performance (Givovich et al., 1994). This suggests that the functions of specific BXDs are genotype- and tissue-dependent. We also observed a positive association between DIMBOA and aphid feeding on phloem sap through their time spent in the phloem phase (duration of E) and a relatively short time spent in the first probing on leaf-1, proposing a different potential role for DIMBOA other than protecting plants against aphids (Hu et al., 2018). Overall, these findings indicate that HDMBOA-Glc/HM2BOA-Glc and DIMBOA might have different transporting abilities from the whole leaf into the phloem; that may affect their role in aphid defense, which requires further investigation.

### Wild Emmer Wheat Germplasm Can Be Used for Improving Aphid-Resistance Traits

The results revealed that both the BXD and trichome density levels depend on the wheat genetic background. Due to the large diversity within the resistance reactions of wild ancestors, wheat progenitors are commonly used as genetic sources for breeding other elite wheat cultivars (Pont et al., 2019). However, the claim that wild ancestors are resources of resistance does not mean that all wild accessions are resistant, but only selected accessions. For example, a recent study that compared the *Metopolophium dirhodum* aphid population of four cultivars of spring bread wheat and two WEW accessions revealed that one of the WEWs, named Rudico, was highly susceptible (Platková et al., 2020). This supported our previous report, where we measured the *R. padi* aphid progeny of three wheat genotypes, two domesticated wheat cultivars (Chinese Spring bread wheat and Svevo) and a WEW named Zavitan, and found that Zavitan is significantly more aphid susceptible than the two domesticated wheat cultivars (Batyrshina et al., 2020b). Thus, for breeding purposes, the wheat progenitors should be carefully screened and chosen to avoid the transmission of undesirable traits such as aphid susceptibility. The WEW panel we used in this research possesses a wide variation in aphid response. The TD-1855 accession had an optimal combination of both BXDs and trichomes and possibly other unrevealed mechanisms that allowed it to be more aphid-resistant to *R. padi* aphids than the other accessions. This accession may be a potential genetic source for enhancing wheat resistance.

## Conclusion

In this study, we investigated the effectiveness of plant physical and chemical defense strategies against insect herbivory. We took advantage of WEW accession diversity and their spatial leaf positions under different conditions (constitutive and aphid-induced) to elucidate the differential mechanisms of plant defense. Our results suggest that physical defense by trichome density was more pronounced in the youngest leaf on which aphids performed poorly, while chemical defense by BXDs showed a complex response at the leaf and phloem level that altered aphid feeding preference. Moreover, we identified a resistant WEW accession that might be used to improve aphid resistance in cultivated wheat. The potential of this WEW accession as an aphid-resistant genetic resource should be further tested in the lab and in the field at various developmental stages and aphid exposure durations.

## Data Availability Statement

The original contributions presented in the study are included in the article/Supplementary Material, further inquiries can be directed to the corresponding author.

## Author Contributions

AS, BD, HS, and VT conceived and designed the experiments and contributed to the writing of the manuscript. AS performed the experiments. AS and VT analyzed the data. All authors contributed to the article and approved the submitted version.

## Conflict of Interest

The authors declare that the research was conducted in the absence of any commercial or financial relationships that could be construed as a potential conflict of interest.

## Footnotes

1 https://imagej.nih.gov/ij/

2 www.jmp.com
