# Supplementary material for: The Effectiveness of Physical and Chemical Defense Responses of Wild Emmer Wheat Against Aphids Depends on Leaf Position and Genotype
Source: Front Plant Sci. 2021 Jun 28;12:667820. doi: 10.3389/fpls.2021.667820 (PMC8273356; doi:10.3389/fpls.2021.667820)
Supplement: Supplementary file 1 [file Data_Sheet_1.pdf]

Figure S1

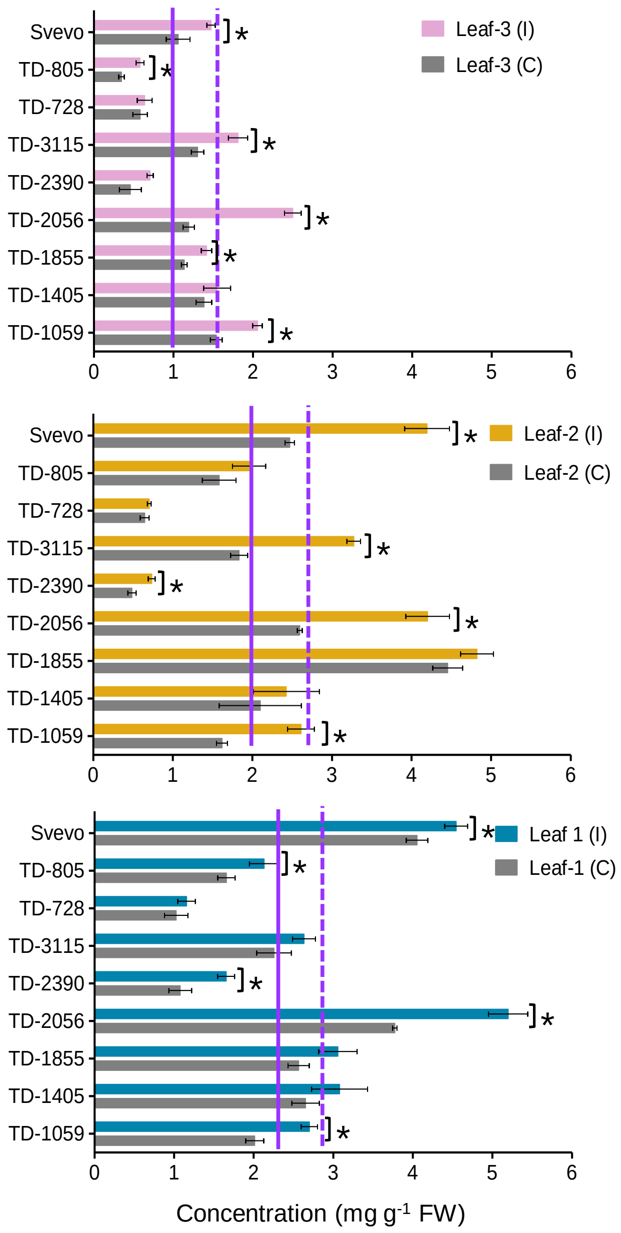

Source	DF	F ratio	*P* value
Leaf position	2, 215	366.40	**p < 0.0001**
Accession	8, 215	173.80	**p < 0.0001**
Leaf position × accession	16, 215	32.17	**p < 0.0001**
Treatment	1, 215	167.81	**p < 0.0001**
Accession × treatment	8, 215	9.07	**p < 0.0001**
Leaf position × treatment	2, 215	5.83	p = 0.0036
Leaf position × accession × treatment	16, 215	1.58	**p = 0.0782**

Figure S1. Quantification of DIM2BOA-Glc content from eight selected WEW accessions and one domesticated durum wheat cultivar. DIM2BOA-Glc contents (mg g^{-1} FW) was evaluated in leaf-1, leaf-2, and leaf-3 both under untreated- control (C) and 96 h following infestation (I) with *R. padi* using the 'whole-cage-choice bioassay'. Values are expressed as mean ± SE (n = 4). The purple solid and dotted line represents the overall mean value on individual leaf type under control and infested conditions among the accessions, respectively. The effect of leaf position, accession, treatment, and their interaction (leaf position × accession × treatment) were tested by two-way ANOVA analysis. Asterisk represents the significant difference between treatments in particular accession analyzed by Student's *t*-tests ($p < 0.05$). In bold, parameters that were significantly affected $p < 0.05$.

Figure S2

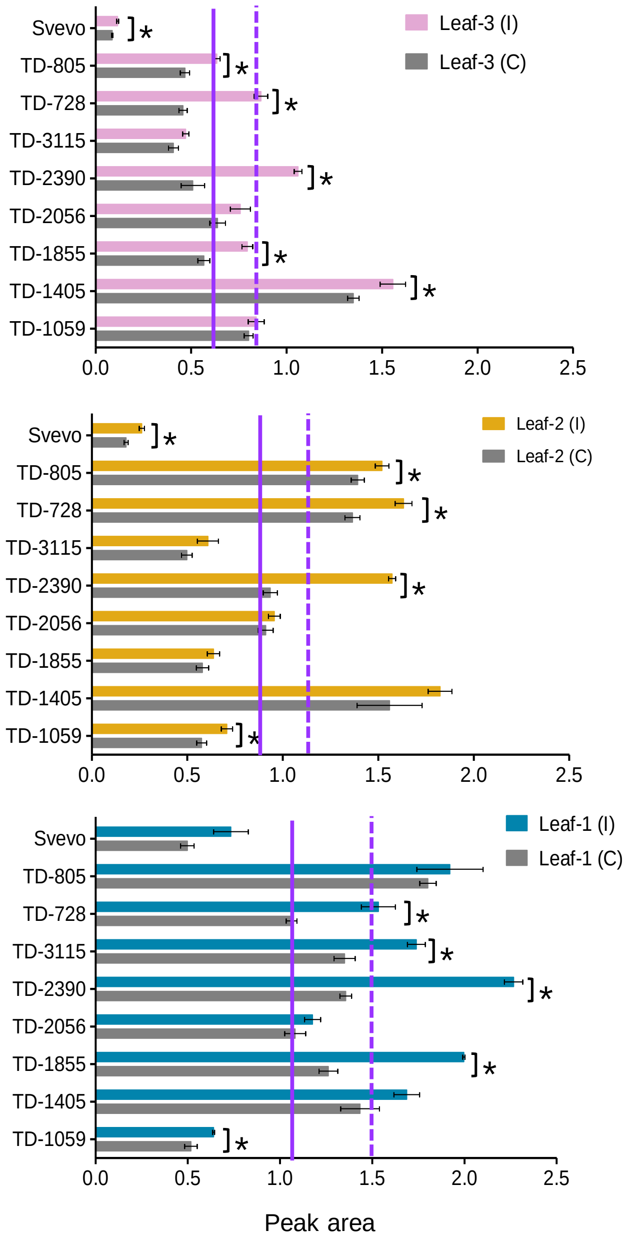

Source	DF	F ratio	*P* value
Leaf position	2, 215	640.72	**p < 0.0001**
Accession	8, 215	282.46	**p < 0.0001**
Leaf position × accession	16, 215	67.44	**p < 0.0001**
Treatment	1, 215	292.72	**p < 0.0001**
Accession × treatment	8, 215	19.73	**p < 0.0001**
Leaf position × treatment	2, 215	15.23	**p < 0.0001**
Leaf position × accession × treatment	16, 215	2.68	**p = 0.0009**

Figure S2. Quantification of HDMBOA-Glc/HM2BOA-Glc content from selected wheat genotypes. HDMBOA-Glc/HM2BOA-Glc contents (peak area) was evaluated in leaf-1, leaf-2, and leaf-3 both under untreated- control (C) and 96 h following infestation (I) with *R. padi* using the 'whole-cage-choice bioassay'. Values are expressed as mean ± SE (n = 4). The purple solid and dotted line represents the overall mean value on individual leaf type under control and infested conditions among the accessions, respectively. The effect of leaf position, accession, treatment and their interaction (leaf position × accession × treatment) were tested by two-way ANOVA analysis. Asterisk represents the significant difference between treatments in particular accession analyzed by Student's *t*-tests ($p < 0.05$). In bold, parameters that were significantly affected $p < 0.05$.

Figure S3

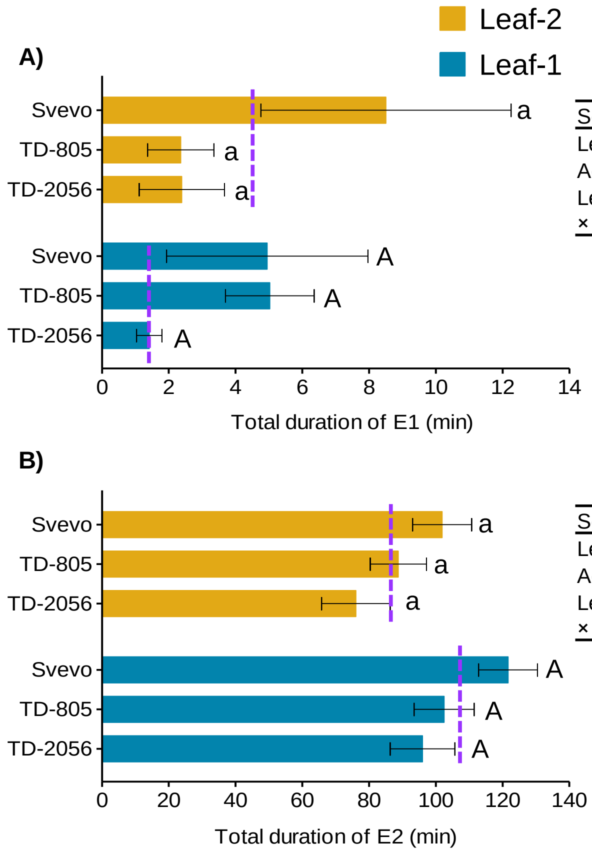

A)

Source	DF	F ratio	P value
Leaf position	1, 107	0.1257	p = 0.723
Accession	2, 107	2.581	p = 0.080
Leaf position × accession	2, 107	1.060	p = 0.350

B)

Source	DF	F ratio	P value
Leaf position	1, 107	5.629	**p = 0.019**
Accession	2, 107	4.008	**p = 0.021**
Leaf position × accession	2, 107	0.0734	p = 0.929

Figure S3. Feeding behavior of R. padi aphid on leaf-1 and leaf-2 of selected wheat genotypes. (A-B) summary of electrical penetration graph (EPG) waveforms recorded in minutes for the total duration of E1 (A) and total duration of E2 (B). Values are expressed as mean ± SE (n = 18). The purple dotted line represents the mean value for each leaf type among the accessions. The effect of leaf position, accession, and their interaction (leaf position × accession) were tested by two-way ANOVA analysis. Significant differences between accessions are indicated by different letters (one way ANOVA, Tukey's honestly significant difference, $p < 0.05$). In bold, parameters that were significantly affected $p < 0.05$.

Figure S4

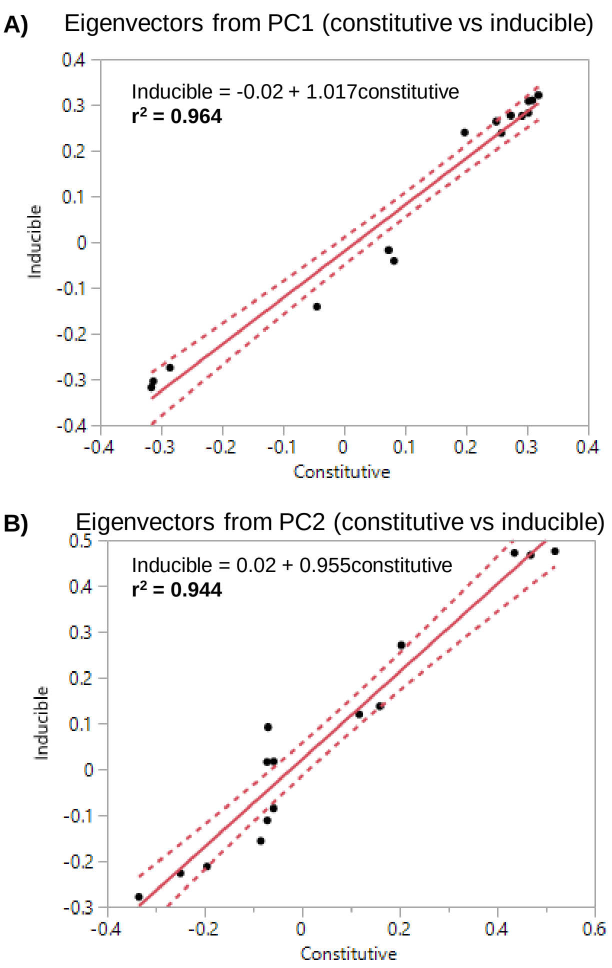

Figure S4. Correlation analysis of eigenvectors from PC1 (A) and PC2 (B) under constitutive and aphid-induced conditions for the physical and chemical defensive traits of selected wheat genotypes.

Figure S5

Summary of Fit

RSquare	0.499287
RSquare Adj	0.469244
Root Mean Square Error	2.45722
Mean of Response	3.785148
Observations (or Sum Wgts)	54

Analysis of Variance

Source	DF	Sum of Squares	Mean Square	F Ratio
Model	3	301.03643	100.345	16.6192
Error	50	301.89644	6.038	**Prob > F**
C. Total	53	602.93286		<.0001*

Parameter Estimates

Term	Estimate	Std Error	t Ratio	Prob>|t|
Intercept	6.3036069	1.293082	4.87	<.0001*
Physical defense	-0.138965	0.021087	-6.59	<.0001*
Chemical defense	-1.464111	0.517534	-2.83	0.0067*
Total defense	3.0835506	1.040881	2.96	0.0047*

Figure S5. Effectiveness of plant physical, chemical (sum of all three BXD), and total defenses (each value [trichomes and each BXD]) against aphid performance. Data were obtained from eight selected WEW accessions and one cultivated durum wheat, normalized to median for multiple linear regression analysis.
